# Inhibitors of Rho kinase (ROCK) signaling revert the malignant phenotype of breast cancer cells in 3D context

**DOI:** 10.18632/oncotarget.9395

**Published:** 2016-05-17

**Authors:** Masahiro Matsubara, Mina J. Bissell

**Affiliations:** ^1^ Life Sciences Division, Lawrence Berkeley National Laboratory, University of California, Berkeley, Berkeley, CA, USA; ^2^ Present address: Oncology Research Laboratories, Kyowa Hakko Kirin Co., Ltd., Japan

**Keywords:** ROCK, polarity, invasiveness, three-dimensional culture, breast cancer

## Abstract

Loss of polarity and quiescence along with increased cellular invasiveness are associated with breast tumor progression. ROCK plays a central role in actin-cytoskeletal rearrangement. We used physiologically relevant 3D cultures of nonmalignant and cancer cells in gels made of laminin-rich extracellular matrix, to investigate ROCK function. Whereas expression levels of ROCK1 and ROCK2 were elevated in cancer cells compared to nonmalignant cells, this was not observed in 2D cultures. Malignant cells showed increased phosphorylation of MLC, corresponding to disorganized F-actin. Inhibition of ROCK signaling restored polarity, decreased disorganization of F-actin, and led to reduction of proliferation. Inhibition of ROCK also decreased EGFR and Integrinβ1 levels, and consequently suppressed activation of Akt, MAPK and FAK as well as GLUT3 and LDHA levels. Again, ROCK inhibition did not inhibit these molecules in 2D. A triple negative breast cancer cell line, which lacks E-cadherin, had high levels of ROCK but was less sensitive to ROCK inhibitors. Exogenous overexpression of E-cadherin, however, rendered these cells strikingly sensitive to ROCK inhibition. Our results add to the growing literature that demonstrate the importance of context and tissue architecture in determining not only regulation of normal and malignant phenotypes but also drug response.

## INTRODUCTION

Rho-associated coiled-coil kinase (ROCK) is an evolutionarily conserved serine/threonine kinase, which interacts with RhoA-GTP [1–4]. In humans, ROCK has two homologs: ROCK1 and ROCK2, the genes of which are located on the 18q11 and 2p24 chromosomal regions, respectively. ROCK1 and ROCK2 share 65% overall identity and 92% in their kinase domain at the amino acid sequence level [1, 5]. Both ROCK1 and ROCK2 play key roles in actin cytoskeleton dynamics, controlling cell shape, cell migration, cell motility, cell proliferation and apoptosis [5, 6]. The RhoA/ROCK pathway is activated by several growth factors, adhesion molecules, integrins, and other biologically active substances when bound to their cognate receptors [5–7]. A number of downstream targets of ROCK regulate the assembly of filamentous actin (F-actin) and actin-myosin contractility [5 6]. Activation of ROCK leads to direct phosphorylation of myosin light chain (MLC) and indirect phosphorylation of MLC through inhibition of myosin phosphatase, these events result in actin-myosin contraction. ROCK also phosphorylates LIM kinase (LIMK) causing activation of LIMK, which inhibits cofilin, an actin-depolymerization factor, ultimately reducing depolymerization of actin filaments [5–7].

Breast cancer is the most common cancer among women. Both maintenance of architecture and function of glandular epithelia depend on the tissue microenvironment, which includes growth factors, extracellular matrix and neighboring stromal cells [8]. During breast cancer progression, tissue polarity is lost and regulation of cell proliferation is abrogated [8]. Acquisition of invasive properties and degradation of the extracellular matrix allow cancer cells to invade into the neighboring tissues, lymphatics, and blood vessels [8]. These alterations eventually enable cancer cells to spread into distant organs and metastasize [8]. mRNA and protein expression of ROCK1, ROCK2 and RhoA are increased in the tissue of breast cancer patients; however, only the increased levels of ROCK1 and RhoA correlate with poor patient prognosis [9–13]. Overexpression of RhoA induces pre-neoplastic transformation in mammary epithelial cells [14]. It has also been reported using breast cancer cell lines that ROCK is involved in cancer cell invasion [15, 16].

Observations made using 2D cultures often miss or contradict physiological or clinical findings, whereas observations from 3D cultures are of clinical relevance [17, 18] thus we chose to study the function of ROCK in breast cell lines using physiologically relevant 3D cultures. In order to analyze ROCK function in epithelial homeostasis, we cultured the cell lines of the HMT3522 breast cancer progression series (nonmalignant S1 and their isogenic malignant counterpart T4-2) within 3D laminin-rich ECM (lrECM) gels [19, 20]. Laminin-111 (Ln1) is one of the principal constituents of basement membrane that plays a central role in maintenance of the differentiation status and morphogenesis in breast tissue [21]. In 3D lrECM, nonmalignant S1 cells form polarized, multi-cellular growth-arrested and luminal-like colonies in response to the Ln-1 in the basement membrane; the S1 colonies resemble the alveolar structures of normal human mammary tissue, referred to as acini. In contrast, under the same conditions, malignant T4-2 cells form disorganized and non-polar colonies that grow continuously [19, 20]. EGF receptor (EGFR), integrin, and aerobic glycolysis -signaling pathways are hyperactivated in malignant T4-2 cells [22–24], and reciprocal interactions among these pathways occur in T4-2 cells when grown in 3D lrECM [22–24]. Importantly, we have demonstrated inhibition of various signaling pathways enables T4-2 cells to form polarized and growth-arrested colonies (without any change in their genotype), and they look similar to the colonies formed by nonmalignant S1 cells [22–25] in a process called “phenotypic reversion.” This 3D lrECM culture system has been applied to many breast epithelial cell lines [26, 27] including MCF10A and MDA-MB-231 cells. The resulting 3D morphology of the colonies is used to phenotypically distinguish malignant from nonmalignant cell lines [26, 27].

We previously demonstrated that T4-2 cells in 3D IrECM show disorganized cytoskeletal F-actin [22, 23] and increased expression of genes involved in cytoskeleton organization [25]. Thus, we postulated that cytoskeletal rearrangement induced by deregulation of RhoA/ROCK pathway is a critical step in breast cancer progression. We found that RhoA, ROCK1, ROCK2 and phosphorylated MLC, which is downstream of the RhoA/ROCK signaling pathway, were increased in T4-2 cells compared with S1 cells. Inhibition of ROCK considerably improved polarity, and suppressed proliferation and invasion in T4-2 cells, while only slightly affecting S1 cells. Inhibition of ROCK caused coordinated down-regulation of the EGFR and integrinβ1 signaling pathways. This down-regulation was evident in 3D cultures, but not observed in 2D cultures, highlighting the importance of studying these pathways under physiologically relevant conditions. Inhibition of ROCK also converted aggressive MDA-MB-231 cells lacking E-cadherin into a less malignant phenotype. The combination of overexpression of E-cadherin and inhibition of ROCK induced apoptosis in MDA-MB-231 cells. These observations are consistent with our hypothesis that RhoA/ROCK signaling contributes to breast cancer progression. Furthermore, these results may provide information in order to develop novel therapeutic targets and strategies for treatment of breast cancer.

## RESULTS

### Expression of ROCK in cultures of 3D lrECM is elevated in malignant T4-2 cells as compared to nonmalignant S1 cells

To understand the role of ROCK signaling in breast cancer, we utilized cell lines from our well-characterized human breast cancer progression series, the HMT 3522 nonmalignant S1 cells and the malignant T4-2 cells [22–25]. Previous work uncovered many aspects of signal integration that allow for the nonmalignant S1 cells to form organized and polar growth arrested colonies when grown in 3D gels of lrECM [22–25]. These signaling pathways have gone awry in the T4-2 cells and in other breast cancer cell lines we have examined [22–27]. Although we know a great deal about how signals from the microenvironment are transduced *via* intracellular signaling, there are still signaling nodes that remain to be investigated in order to completely ‘close the loop’ on how an acinus is formed and maintained within breast tissue.

**Figure 1:**
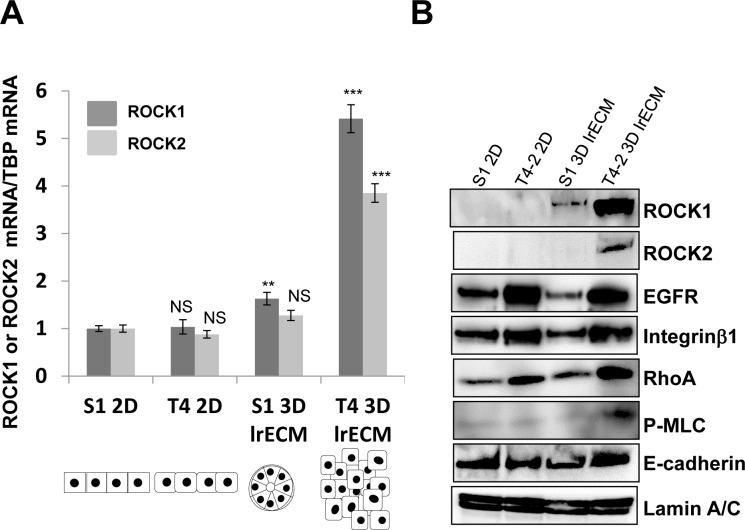
Elevated expression of ROCK1, ROCK2 and RhoA in malignant T4-2 cells in three-dimensional laminin-rich ECM (3D lrECM) **A.** mRNA expression of ROCK1 and ROCK2 in S1 and T4-2 cells monolayer (2D) and 3D lrECM culture were analyzed by real-time quantitative Reverse Transcription PCR (RT-PCR) with specific primer sets. mRNA expression level of ROCK1 and ROCK2 were normalized to that of TATA binding protein (TBP). Values represent means ± SE of six experiments. ROCK1; N.S. (not significant), **P < 0.01, ***P < 0.001 compared with S1 2D group (Student's t). ROCK2; N.S. (not significant), ***P < 0.001 compared with S1 2D group (Student's t). The Illustration of morphologies of S1 and T4-2 cells in 2D and 3D is shown in the bottom. **B.** Protein expression of ROCK1, ROCK2, EGF receptor (EGFR), Integrinβ1, RhoA, phosphorylated myosin light chain (P-MLC), E-cadherin and Lamin A/C in nonmalignant S1 cells and malignant T4-2 cells in 2D and 3D lrECM cultures. Total cell lysates were analyzed by Western blotting with their specific antibodies.

ROCK and RhoA are within a signaling pathway that is often misregulated in breast cancer progression [9–16]. Thus, we examined the expression of ROCK and RhoA in nonmalignant S1 cells and their counterpart malignant T4-2 cells using monolayer plastic (2D) culture and 3D lrECM gel culture. Immunoblot showed that T4-2 cells produce higher amounts of Integrinβ1 and EGFR as compared to S1 cells, irrespective of whether cultured in 2D or 3D lrECM culture (Figure 1B). These observations were consistent with previous results from our laboratory [22–25]. Expressions of both ROCK1 and ROCK2 in 2D culture were barely detectable and were similar between S1 and T4-2 cells but levels of ROCK1 and ROCK2 were substantially elevated in T4-2 cells grown in 3D lrECM. Expression pattern of RhoA, which is an upstream effector of ROCK, was similar to that of Integrinβ1 and EGFR in S1 and T4-2 cells, in that the levels of RhoA were higher in T4-2 cells regardless of whether cells were cultured in 2D or 3D (Figure 1B). Quantification of ROCK1 and ROCK2 mRNAs corroborated results from the immunoblot (Figure 1A). ROCK directly and indirectly phosphorylates myosin light chain (MLC), leading to actin-myosin contraction [1, 5–7] and we found phosphorylated MLC was especially enhanced in T4-2 cells in 3D lrECM (Figure 1B), suggesting that RhoA/ROCK signaling is indeed activated in T4-2 cells grown in 3D lrECM. Our observations using our physiologically relevant 3D culture system are consistent with several studies using clinical samples of breast cancer, which have shown expression of RhoA and ROCK1 are upregulated in the tumor tissue [9–13], thus, supporting the use of this culture system for the investigation of ROCK signaling in breast cancer progression.

### RhoA/ROCK activity correlates with disrupted acinar architecture of breast cancer cells grown in 3D lrECM

**Figure 2:**
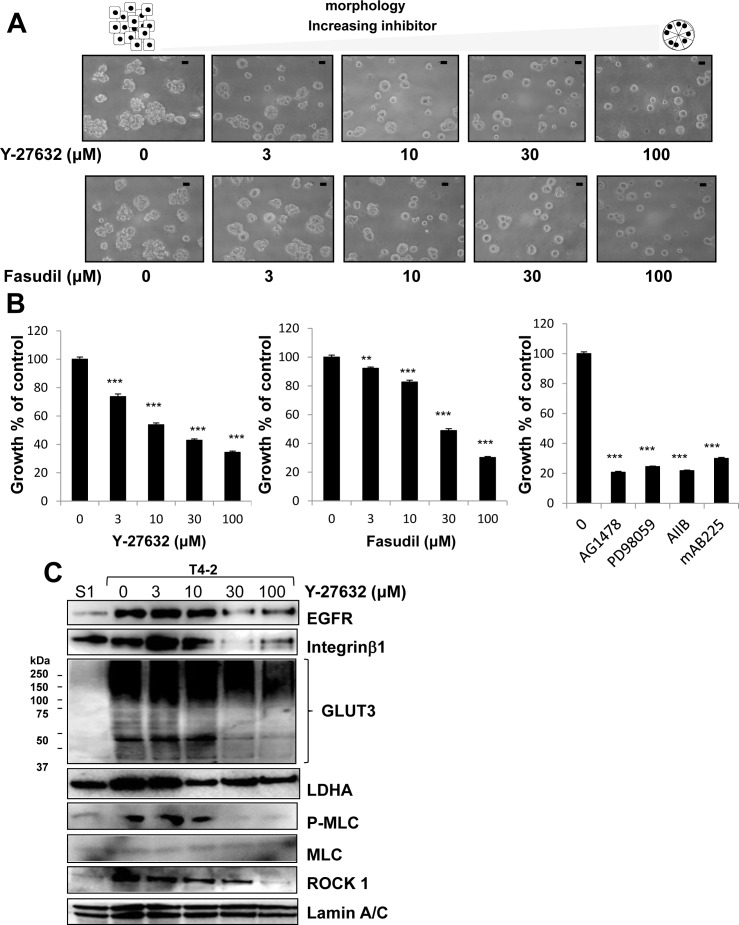
Involvement of ROCK in disorganized phenotype of malignant T4-2 cells in 3D lrECM **A.** Morphological alternations of T4-2 cells treated with ROCK inhibitor (Y-26732 or Fasudil, 3~100 μM). Scale bars: 20 μm. **B.** Inhibition of proliferation of T4-2 cells treated with ROCK inhibitor (Y-26732 or Fasudil, 3~100 μM), EGFR inhibitor (AG1478, 0.1 μM), MEK inhibitor (PD98059, 10 μM), Integrinβ1 function blocking antibody (AIIB, 100 μg/mL) or EGFR function blocking antibody (mAB225, 4 μg/mL). Cell viability was assessed by MTT assay. Values represent means ± SE of four experiments. **P < 0.01, ***P < 0.001 in the upper graph compared with vehicle control group (Dunnett). ***P < 0.001 in the lower graph compared with vehicle control group (Student's t). **C.** Down-regulation of EGFR, Integrinβ1, glucose transporter 3 (GLUT3), lactate dehydrogenase A (LDHA), P-MLC and ROCK1 in T4-2 cells treated with ROCK inhibitor (Y-26732, 3~100 μM). Total cell lysates were analyzed by Western blotting with their specific antibodies.

**Figure 3:**
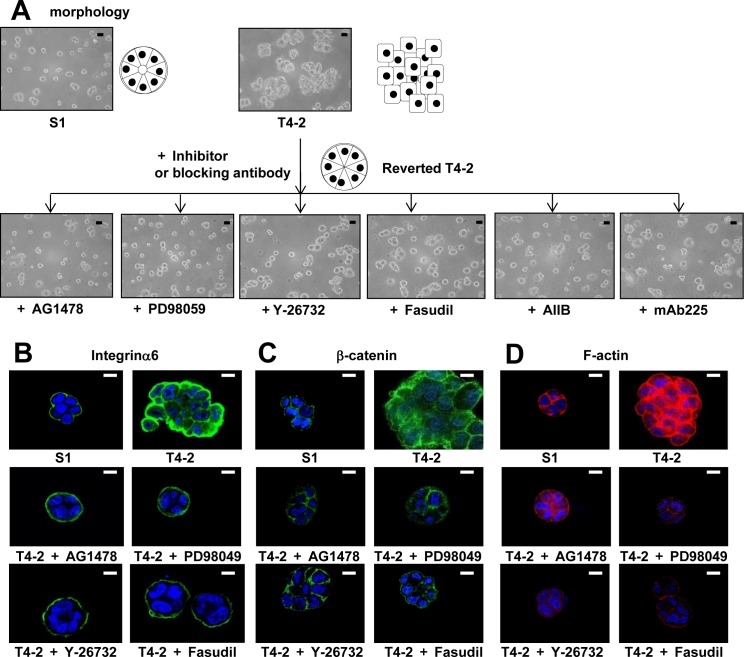
Involvement of ROCK in loss of polarity and actin cytoskeletal rearrangement of T4-2 cells in 3D lrECM **A.** Morphological alternations of T4-2 cells treated with ROCK inhibitor (Y-26732 or Fasudil, 30 μM), EGFR inhibitor (AG1478, 0.1 μM), Integrinβ1 function blocking antibody (AIIB, 100 μg/mL), EGFR function blocking antibody (mAB225, 4 μg/mL) or MEK inhibitor (PD98059, 10 μM). Scale bars: 20 μm. **B. C.** and **D.** Confocal immunofluorescence of basal maker, lateral marker and cytoskeletal filamentous**F.**-actin. Their images were obtained using their specific antibodies (**B.** Integrinα6; Green, **C.** β-catenin; Green) and Alexa Fluor-conjugated phalloidin (**D.**) F-actin; Red). Nuclei were stained with DAP (Blue). Scale bars: 10 μm.

We previously utilized this 3D lrECM gel culture assay to identify the involvement of several signaling pathways, such as EGFR, integrin and glucose metabolism, among others [22–25]. Moreover, inhibition or normalization of signaling pathways leads to phenotypic reversion of breast tumor cells, where T4-2 cells are able to form acinar-like structures which are similar to the nonmalignant S1 cells [22–25]. To evaluate functional involvement of ROCK in the malignant phenotype of T4-2 cells, we applied two different ROCK inhibitors Y-27632 [28] and Fasudil [29], which are well-characterized specific ROCK inhibitors with differing chemical structure [1, 5, 6]. Both inhibitors suppress the function of ROCK1 and ROCK2 by inhibition of kinase activity in a competitive manner with ATP [1, 5, 6, 30, 31]. We found that these two ROCK inhibitors reduced the disorganized phenotype of T4-2 cells in 3D lrECM in a concentration-dependent manner (3~100 μM) (Figure 2A). To determine if the ROCK inhibitors decreased proliferation, cells were extracted from the lrECM gel culture and the protein concentration of the cells was used as a surrogate marker for proliferation. We found the protein concentration of T4-2 cells treated with ROCK inhibitors was decreased (data not shown). Both ROCK inhibitors suppressed cell proliferation of T4-2 cells in a manner comparable to the EGFR inhibitor (AG1478, 0.1 μM), Integrinβ1 blocking antibody (AIIB, 100 μg/mL), EGFR blocking antibody (mAB225, 4 μg/mL) or the MEK inhibitor (PD98059, 10 μM) (Figure 2B), all of which were shown to revert the malignant phenotype of T4-2 cells [22, 23]. The higher concentration of ROCK inhibitors was required to suppress cell proliferation in comparison with the concentration of EGFR inhibitor and MEK inhibitor (Figure 2B). The ROCK inhibitor (Y-26732) decreased phosphorylation of MLC, which is a downstream target of ROCK (Figure 2C). In the same concentration range where the reduction of MLC phosphorylation was observed, we found down-modulation of the levels of EGFR and Integrinβ1 (Figure 2C), which we previously showed are hallmarks for T4-2 cells phenotypic reversion [23]. We recently reported that levels of GLUT3 (the facilitative glucose transporter 3 encoded by SLC2A3), lactate dehydrogenase A (LDHA) and other aerobic glycolysis-related proteins, are increased in T4-2 cells and that the high levels of GLUT3 contribute to the malignant phenotype [24]. Inhibition of ROCK also reduced the amount of GLUT3 and LDHA proteins (Figure 2C). Interestingly, we found that inhibition of ROCK showed a decrease in ROCK1 protein itself (Figure 2C). The reverted morphological phenotype of T4-2 cells resulting from ROCK inhibition was similar to the phenotype observed by inhibition of EGFR, Integrinβ1 or MEK in T4-2 cells and similar to the phenotype of nonmalignant S1 cells (Figure 3A). S1 cells in 3D lrECM form polarized colonies, as indicated by the basal location of integrinα6, localized β-catenin at the lateral cell-cell junction and well-organized F-actin (Figure 3B, 3C and 3D). Inhibition of ROCK exhibited improvement of basal polarity and β-catenin localization, and the decrease in disorganized F-actin in T4-2 cells (Figure 3B, 3C and 3D). We also observed that another ROCK inhibitor (GSK-429286, [32]) induced the reversion of phenotype of T4-2 cells (data not shown). These data suggest that an overactive ROCK pathway leads to an impairment in the ability of breast cells to form and maintain an organized, polar and quiescent acinar structure, and contributes to the malignant phenotype of breast epithelial cells.

### The RhoA/ROCK pathway integrates with other signaling pathways disrupted in T4-2 cells and gives rise to the malignant phenotype

**Figure 4:**
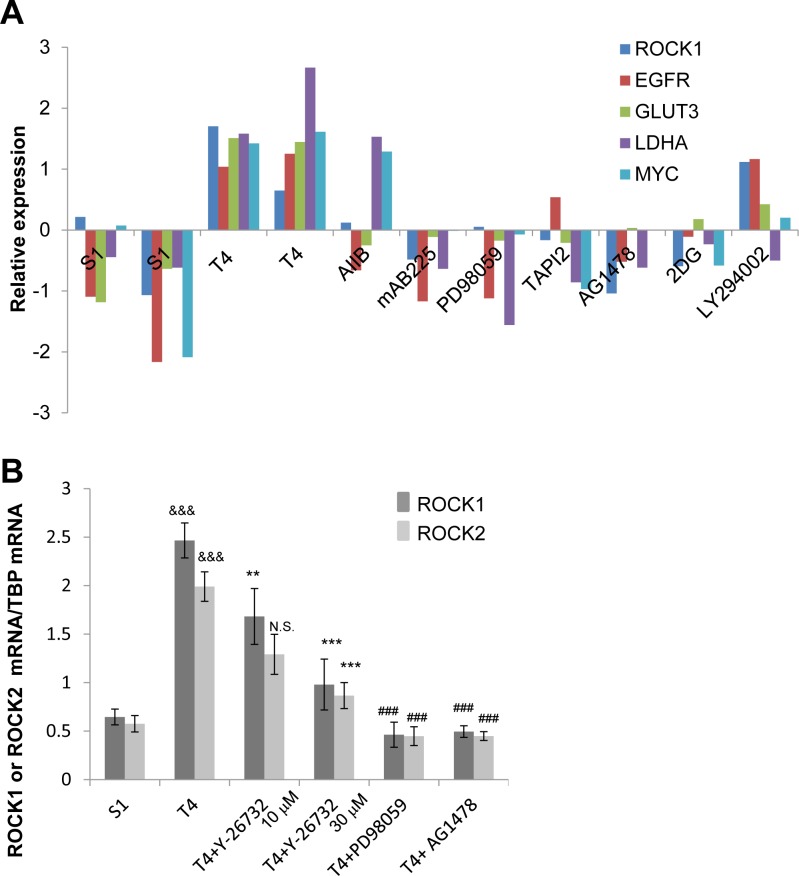
Downregulation of ROCK in reverted T4-2 cells in 3D lrECM **A.** mRNA expression of ROCK1, EGFR, GLUT3, LDHA and c-MYC in S1, and T4-2 and reverted T4-2 cells. Data were retrieved from microarray analysis done by our laboratory and shown as relative expression after preprocessing, normalizing and filtering (arbitrary units). The array data have been stored in GEO (Accession no. GSE50444). AIIB; Integrinβ1 function blocking antibody, mAB225; EGFR function blocking antibody, PD98059; MEK inhibitor, TAPI2; TNF-α converting enzyme (TACE), ADAM (a disintegrin and metalloproteinases) and matrix metalloproteinase (MMP) inhibitor, AG1478; EGFR inhibitor, 2DG (2-deoxyglucose); glucose metabolism inhibitor, LY294002; phosphoinositide 3-kinase inhibitor. **B.** mRNA expression of ROCK1 and ROCK2 in S1, T4-2, and T4-2 cells treated with ROCK inhibitor (Y-26732, 10, 30 μM), MEK inhibitor (PD98059, 10 μM) and EGFR inhibitor (AG1478, 0.1 μM) were analyzed by real-time quantitative RT-PCR. mRNA expression levels of ROCK1 and ROCK2 were normalized to that of TBP. Values represent means ± SE of six experiments. ROCK1; ^&&&^*P* < 0.001 compared with S1 group (Student's t). **P < 0.01, ***P < 0.001 compared with vehicle control group (Dunnett). ^###^ *P* < 0.001 compared with vehicle control group (Student's t). ROCK2; ^&&&^*P* < 0.001 compared with S1 group (Student's t). N.S. (not significant), ***P < 0.001 compared with vehicle control group (Dunnett). ^###^ *P* < 0.001 compared with vehicle control group (Student's t).

We previously compared gene expression profiles of S1 cells, T4-2 cells and T4-2 cells phenotypically reverted by a number of inhibitors and blocking antibodies [25]. We identified an increase in expression of genes involved in actin organization in T4-2 cells using gene set enrichment analysis [25]. Here, we revisited the microarray data and found that ROCK1 mRNA was increased in T4-2 cells compared with S1 cells (Figure 4A). In addition, ROCK1 mRNA expression was commonly down-regulated in the reverted T4-2 cells just as we observed the down-regulation of the mRNA for EGFR, GLUT3, LDHA and c-MYC (Figure 4A). Using quantitative PCR, we confirmed that mRNA of ROCK1 and ROCK2 were decreased in T4-2 cells treated with ROCK inhibitor (Y-26732), MEK inhibitor (PD98059) or EGFR inhibitor (AG1478) (Figure 4B).

**Figure 5:**
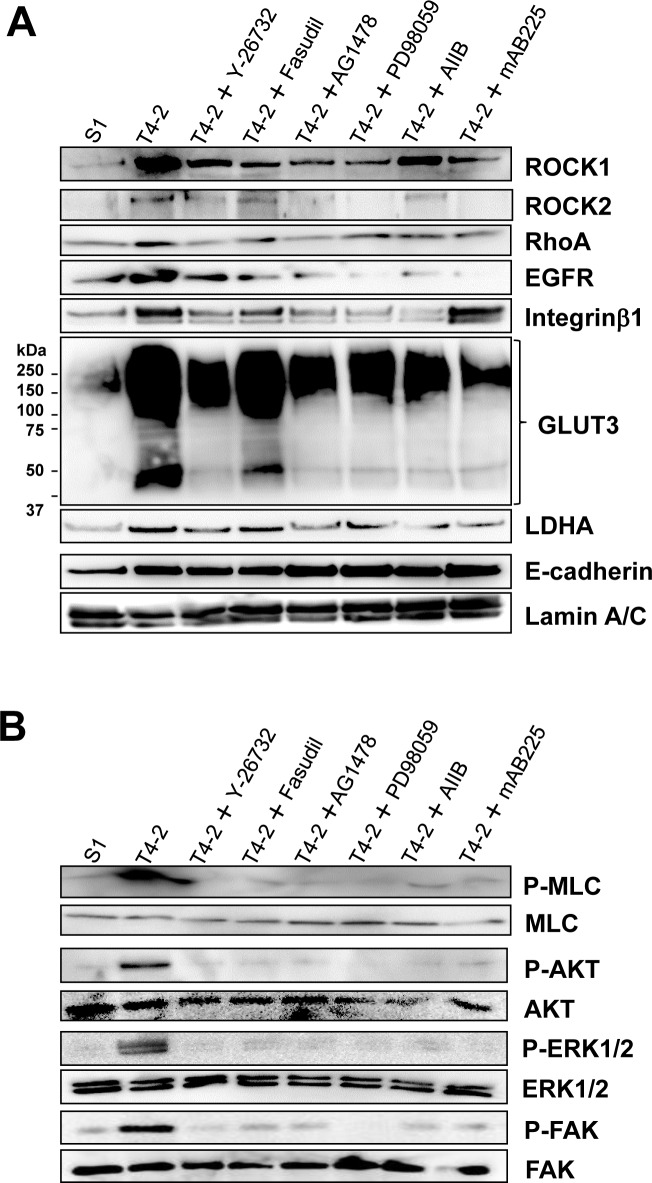
Reciprocal interaction of ROCK and other signaling pathways in T4-2 cells in 3D lrECM **A.** Protein expression of ROCK1, ROCK2, RhoA, EGFR, Integrinβ1, GLUT3, LDHA, E-cadherin and Lamin A/C **B.** Phosphorylated (shown as P-) and total protein level of MLC, Akt, ERK1/2, FAK. Total cell lysates were analyzed by Western blotting with their specific antibodies. T4-2 cells in 3D lrECM were treated with ROCK inhibitor (Y-26732, 30 μM), ROCK inhibitor (Fasudil, 30 μM), EGFR inhibitor (AG1478, 0.1 μM), MEK inhibitor (PD98059, 10 μM), Integrinβ1 function blocking antibody (AIIB, 100 μg/mL) or EGFR function blocking antibody (mAB225, 4 μg/mL).

Based on this information, we hypothesized that the other signaling pathways integrate or modulate RhoA/ROCK signaling in T4-2 cells and vice versa. To test this, we investigated protein expression and phosphorylation of key molecules. Protein expression of RhoA, ROCK1 and ROCK2 was decreased by treatment with EGFR inhibitor (AG1478, 0.1 μM), MEK inhibitor (PD98059, 10 μM), Integrinβ1 function blocking antibody (AIIB, 100 μg/mL) and EGFR function blocking antibody (mAB225, 4 μg/mL) (Figure 5A), and consequently reduced phosphorylation level of MLC in T4-2 cells (Figure 5B). Two ROCK inhibitors (Y-27632 and Fasudil, 30 μM) caused a decrease in both EGFR and Integrinβ1 protein levels (Figure 5A), also as demonstrated above (Figure 2D). In accordance with the decrease in these receptor proteins, phosphorylation levels of AKT, ERK1/2 and FAK, which are downstream molecules of EGFR and Integrinβ1, were reduced when ROCK was inhibited (Figure 5B). Furthermore, these reversion agents, including the ROCK inhibitor Y-26735, reduced the levels of GLUT3 and LDHA proteins in T4-2 cells (Figure 5A). These observations suggest that the ROCK signaling pathways are integrated with other signaling pathways previously investigated in the laboratory and found to be essential signaling nodes for correct acinar formation. Disruption of these pathways leads to a malignant phenotype observed in the T4-2 cells and other breast cancer cells.

Inhibition of ROCK reduced actin-stress fibers, and induced morphological changes in T4-2 cells grown in 2D (Figure S1A). In spite of phenotypic changes, EGFR, Integrinβ1, GLUT3, phosphorylated ERK1/2 and LDHA were not down-modulated by ROCK inhibitor in the 2D cultures (Figure S1B). This contrasts with observation that these proteins were down-regulated by inhibition of ROCK in 3D (Figure 2C, Figure 5A and Figure 5B), indicating that the events causing the morphological changes observed in 2D culture are insufficient or not integrated correctly in order to down-regulate cross-signaling among these pathways that we have previously identified as critical for acinar formation. These results also support our hypothesis that cell-ECM interaction and/or 3D structure is crucial for reciprocal interaction among these signaling pathways.

### Increased ROCK activity disrupts the nonmalignant phenotype of breast epithelial cells

We further examined effects of ROCK function in nonmalignant S1 cells since these cells express less RhoA and ROCK proteins than T4-2 cells (Figure 1A). S1 cells cultured in 3D lrECM showed proper localization of the basal and basolateral marker Integrinα6 and β-catenin, respectively, exhibited organized F-actin and formed acinus-like structure (Figure S2A). In contrast to T4-2 cells, two ROCK inhibitors (Y-27632 and Fasudil, 30 μM) had no apparent effects on these phenotypic parameters in S1 cells (Figure S2A). Inhibition of ROCK only moderately affected cell proliferation (Figure S2B). These inhibitors did not induce down-regulation of protein expression of EGFR, Integrinβ1, GLUT3, phosphorylated ERK1/2 and LDHA (Figure S2C). These moderate effects of ROCK inhibition are most likely attributed to a less activated RhoA/ROCK signaling pathway in S1 cells as compared to T4-2 cells, as described above (Figure 1).

**Figure 6:**
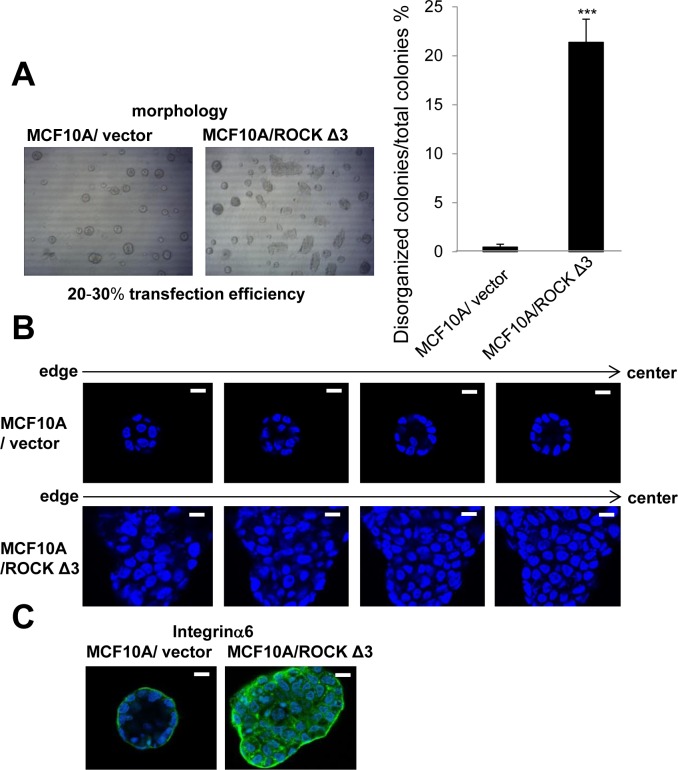
Effects of overexpression of constructively active ROCK on nonmalignant MCF10A cells in 3D lrECM **A.** Morphology of MCF10A transfected with vector or pCAG-myc-ROCKΔ3 expressing constructively active ROCK. Percentage of disorganized colonies per total colonies was calculated using the amount of 4 independent images in each well. Values represent means ± SE of five determinations. ***P < 0.001 compared with vector control group (Student's t). **B.** Representative confocal images of DAPI-stained vector and ROCKΔ3 expressed MCF10A colonies. An arrow shows the direction from edge (left) to center (right) of colony. White scale bars: 10 μm. **C.** Confocal immunofluorescence of basal maker were obtained using anti-Integrinα6 antibody (Green).

We also investigated effects of increased ROCK activity on nonmalignant breast cells using the MCF10A cell line. Like S1 cells, MCF10A cells form polarized luminal structures (Figure 6). Transfection of MCF10A cells with ROCKΔ3, which is known as a constitutively active ROCK mutant [33], significantly increased the number of disorganized colonies compared with vector control when cultured in 3D lrECM gels (Figure 6A). The number of disorganized colonies of MCF10A cells transfected with ROCKΔ3 was reasonable in the sense that the transfection efficiency was 20-30 percent using EGFP-expressing plasmid (data not shown). In addition, the disorganized colonies of MCF10A cells transfected with ROCKΔ3 showed no lumen and loss of polarity (Figure 6B and 6C), suggesting that hyperactivation of ROCK activity disrupts the ability of the epithelial cells to form correct structural organization.

### Effects of inhibition of ROCK on growth in co-cultured T4 cells with lung fibroblasts in IrECM

**Figure 7:**
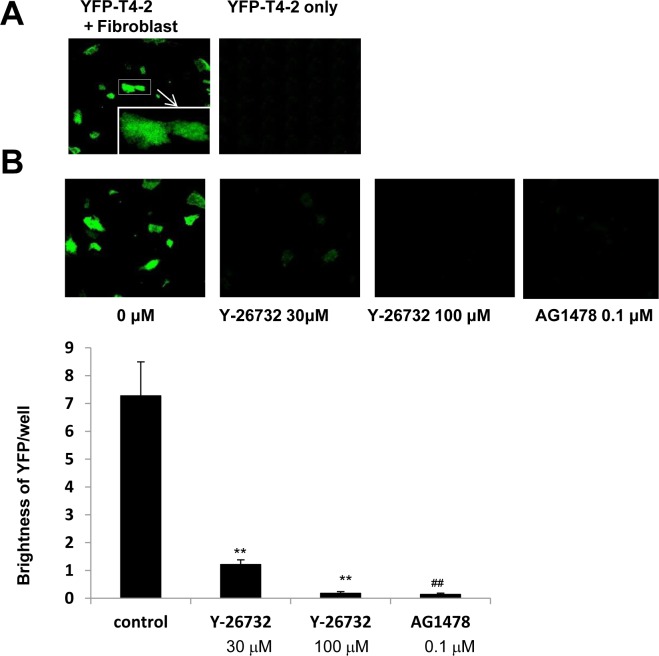
Involvement of ROCK in growth of T4-2 cells co-cultured with fibroblasts in IrECM **A.** In the presence (left panel) or absence (right panel) of cultured human lung fibroblats, YFP-T4-2 cells were plated in unsupplemented DMEM/F12, and then IrECM was dropped into the co-culture system. **C.** YFP-T4-2 cells co-cultured with human lung fibroblasts were treated with ROCK inhibitor (Y-26732, 30 and 100 μM) or EGFR inhibitor (AG1478, 0.1 μM). The brightness of YFP in T4-2 cells per well was measured. Values represent means ± SE of six experiments. **P < 0.01 compared with vehicle control group (Dunnett). ^##^ *P* < 0.01 compared with vehicle control group (Student's t).

If breast cancer cells dissolve ECM components and spread into distant tissues such as lung and bone marrow, they manage to survive and grow under microenvironment containing fibroblasts, mesenchymal stem cells and other type of cells [8]. We implemented co-culture of T4-2 cells with fibroblasts using IrECM, and examined effects of ROCK inhibition on T4-2 cells in this co-culture system. Whereas the starved medium without fibroblasts was not enough for growth of T4-2 cells, T4-2 cells were able to grow in the presence of cultured lung fibroblasts (Figure 7A). Inhibition of ROCK (Y-27632, 30 μM) and EGFR inhibitor (AG1478, 0.1 μM) reduced proliferation in co-cultured T4-2 cells (Figure 7B) while these reagents did not kill lung fibroblasts (data not shown). These observations suggested that ROCK inhibitor may be beneficial for prevention of metastasis.

### Elevated levels of ROCK correlate with the disorganized phenotype in triple negative MDA-MB-231

**Figure 8:**
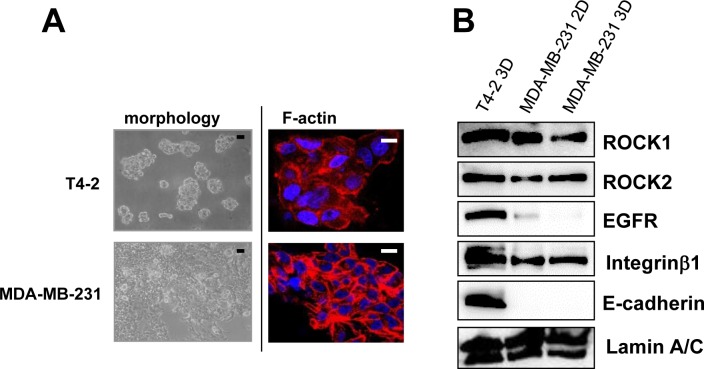
High amount of ROCK in aggressive breast cancer cell line MDA-MB-231 **A.** Morphology and cytoskeletal actin filaments (F-actin; Red, Nuclei; Blue) of T4-2 cells and MDA-MB-231 in 3D lrECM. Black scale bars: 20 μm, White scale bars: 10 μm. **B.** Protein expression of ROCK1, ROCK2, EGFR, Integrinβ1, E-cadherin and Lamin A/C in 2D and 3D lrECM culture. Total cell lysates were analyzed by Western blotting with their specific antibodies.

**Figure 9:**
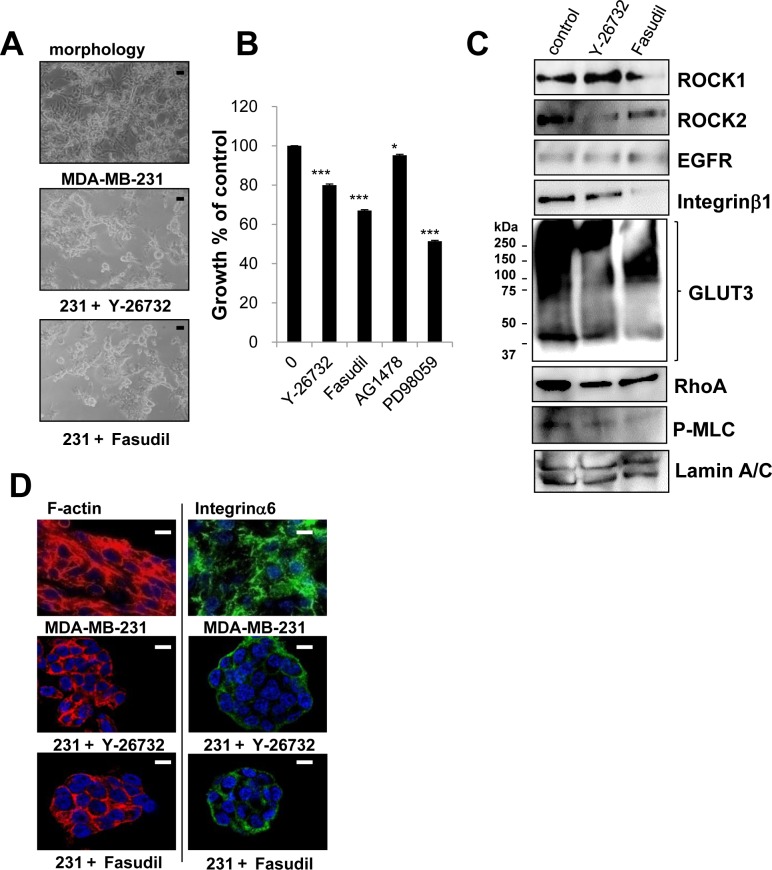
Involvement of ROCK in disorganized phenotype of MDA-MB-231 cells in 3D lrECM **A.** Morphological alternations of MDA-MB-231 cells treated with ROCK inhibitor (Y-26732 or Fasudil, 30 μM). Scale bars: 20 μm. **B.** Partial inhibition of proliferation of MDA-MB-231 cells treated with ROCK inhibitor (Y-26732 or Fasudil, 30 μM), EGFR inhibitor (AG1478, 0.1 μM) and MEK inhibitor (PD98059, 10 μM). Cell viability was assessed by MTT assay. Values represent means ± SE of four experiments. *P < 0.05, ***P < 0.001 compared with vehicle control group (Student's t). **C.** Protein expression of ROCK1, ROCK2, EGFR, Integrinβ1, GLUT3, RhoA, P-MLC and Lamin A/C in MDA-MB-231 cells. Total cell lysates were analyzed by Western blotting with their specific antibodies. **D.** Confocal immunofluorescence images of MDA-MB-231 cells treated with ROCK inhibitor (Y-26732 or Fasudil, 30 μM). Their images were obtained using specific antibody or Alexa Fluor-conjugated phalloidin (Integrinα6; Green, F-actin; Red, Nuclei; Blue). Scale bars: 10 μm.

We previously reported the morphological phenotypes of 25 breast cell lines when grown in identical 3D culture conditions. According to their morphologies, those cell lines were classified into four groups [27]. Of these four groups, stellate types, including MDA-MB-231, are more invasive and proliferative in 3D culture [27]. Based on the fact that MDA-MB-231, a well-characterized triple negative breast cancer cell line, is invasive, metastatic and lacks E-cadherin [26], MDA-MB-231 cells were chosen for further analysis. In agreement with previous findings [26, 27], MDA-MB-231 cells showed more disruptive phenotypes (morphology and F-actin) than T4-2 cells in 3D (Figure 8A). We determined expression of ROCK in MDA-MB-231 cells grown in 2D or 3D cultures. MDA-MB-231 cells grown in 2D culture had high levels of both ROCK1 and ROCK2 proteins, which were comparable to those were observed when T4-2 cells were grown in 3D lrECM (Figure 8B). Next, we examined effects of ROCK inhibition on MDA-MB-231 cells. MDA-MB-231 cells treated with ROCK inhibitor (Y-27632 and Fasudil, 30 μM) formed clusters that phenotypically appeared less aggressive (Figure 9A) [27]. Both ROCK inhibitors also decreased proliferation of MDA-MB-231 cells in 3D IrECM (Figure 9B). However, the inhibition of proliferation was less potent in MDA-MB-231 cells compared to T4-2 cells. Inhibition of ROCK in MDA-MB-231 cells reduced the phosphorylation level of MLC as well as the expression of Integrinβ1, GLUT3, ROCK and RhoA proteins (Figure 9C). Inhibition of ROCK in MD-MBA-231 cells showed significant reduction of disorganized actin-stress fibers, and restored basal polarity (Figure 9D).

We further addressed whether inhibition of ROCK downstream molecule involved in actomyosin contractibility could alter properties of both T4-2 and MDA-MB-231 cells to be less aggressive. The morphological changes induced by Blebbistatin (20 μM), a Myosin II ATPase inhibitor, were similar to those by ROCK inhibitor (Figure S3A). Blebbistatin also inhibited the proliferation (data not shown), restored the basal polarity, and reduced the disorganized actin-stress fibers in both cell lines (Figure S3B). These results suggest increased actomyosin contractibility through activation of ROCK leads to a loss of polarity in both T4-2 and MDA-MB-231 cells in 3D culture.

### Expression of E-cadherin renders MDA-MB-231 cells sensitive to ROCK inhibitors

**Figure 10:**
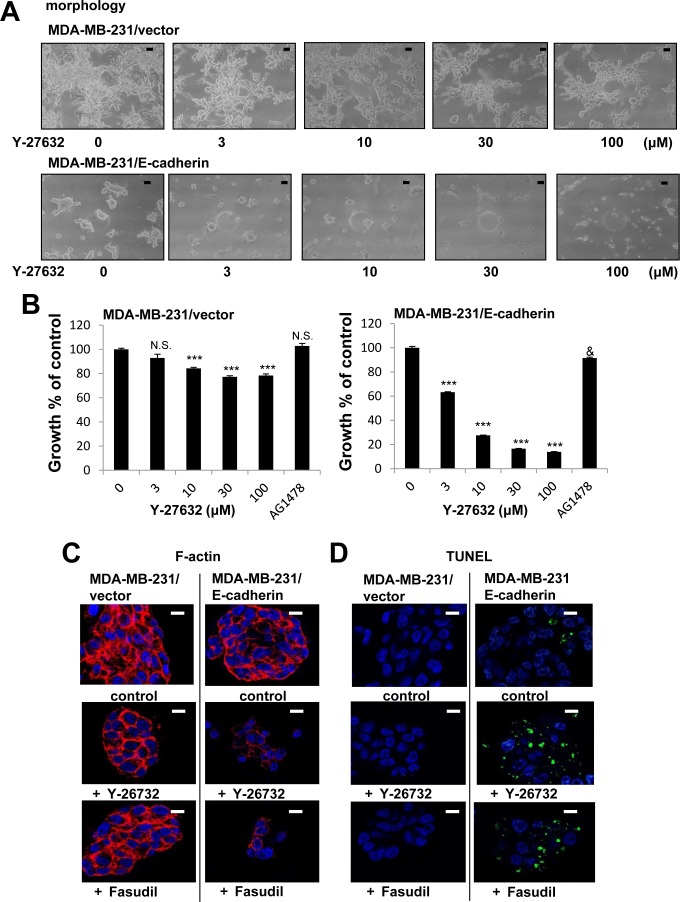
Enhanced growth inhibition by a combination of E-cadherin overexpression and ROCK inhibition in MDA-MB-231 cells in 3D lrECM **A.** Morphological alternations of vector-or E-cadherin-expressing (E-cad-) MDA-MB-231 cells treated with ROCK inhibitor (Y-26732, 3~100 μM). Scale bars: 10 μm. **B.** Inhibition of proliferation of vector-or E-cad-MDA-MB-231 cells treated with ROCK inhibitor (Y-26732, 3~100 μM) and EGFR inhibitor (AG1478, 0.1 μM). Cell viability was assessed by MTT assay. Values represent means ± SE of four experiments. N.S. (not significant), ***P < 0.001 compared with vehicle control group (Dunnett). N.S. (not significant), ^&^*P* < 0.05 compared with vehicle control group (Student's t). **C.** Confocal immunofluorescence of F-actin in vector-or E-cad -MDA-MB-231 cells treated with ROCK inhibitor (Y-26732 or Fasudil, 30 μM). Images were obtained using Alexa Fluor-conjugated phalloidin (F-actin; Red, Nuclei; Blue). Scale bars: 10 μm. **D.** DNA fragmentation in vector-or E-cad-MDA-MB-231 cells treated with ROCK inhibitor (Y-26732 or Fasudil, 30 μM). TUNEL (TdT-mediated dUTP nick end labeling) staining was performed (Fragmented DNA; Green, Nuclei; Blue). Scale bars: 10 μm.

MDA-MB-231 cells are different from T-4 cells in that they lack E-cadherin (Figure 8B). We have previously observed that presence and correct function of E-cadherin is essential for the formation and maintenance of acinar structures as the function-blocking antibody disrupts polarity of S1 cells in 3D culture [34]. Furthermore, we showed that exogenous expression of E-cadherin in MDA-MB-231 cells allows the formation of compact clusters and enhances responsiveness to several reverting agents [26]. These results reinforce the evidence that E-cadherin plays a critical role in epithelial architecture. We used E-cadherin-expressing MDA-MB-231 cells to investigate effects of ROCK inhibition on the phenotype of MDA-MB-231 cells. Expression of ROCK protein was not changed by overexpression of E-cadherin (Figure S4). MDA-MB-231 infected with an empty vector showed the stellate morphology (Figure 10A), and inhibition of ROCK led to less disorganized phenotype (Figure 10A, 10C and 10D), which was similar to the results of parent cells (Figure 9). In contrast, E-cadherin-expressing (E-cad) MDA-MB-231 cells showed less stellate clusters (Figure 10A). Inhibition of ROCK strongly decreased proliferation of E-cad-MDA-MB-231 cells (Figure 10B). On the other hand, EGFR inhibitor (AG1478) barely affected the proliferation of control-and E-cad-MDA-MB-231 cells (Figure 10B), which is consistent with previous observations of MDA-MB-231 cells lacking proliferative response to EGF [35]. We found that E-cad-MDA-MB-231 cells treated with ROCK inhibitor no longer stained with phalloidin (Figure 10C), and appeared to be dead (Figure 10A). Furthermore, a TUNEL assay showed that fragmented DNA was increased by inhibition of ROCK in E-cad-MDA-MB-231 cells, but not in vector-control cells (Figure 10D), suggesting that inhibition of ROCK induced apoptosis in the E-cad-MDA-MB-231 cells. Together, our results suggest that increased ROCK signaling plays a critical role in breast cancer progression. Understanding the role of the ROCK signaling node will be beneficial for the design of new and complementary treatment strategies for breast cancer.

## DISCUSSION

Several studies have shown that elevated expression of RhoA and ROCK1 in breast cancer tissues is associated with poor prognosis [9–13]. Similarly, higher expression of RhoA, ROCK1 and ROCK2 has been found in other types of cancer [36–40]. There are additional reports of somatic mutations of ROCK1 which lead to uninterrupted kinase activity due to the absence of auto-inhibition, resulting in cytoskeletal rearrangements in a number of cancers including breast cancer [41, 42]. In this study, we used physiologically relevant 3D cell culture to assess the mechanism by which ROCK activity participates in malignant progression. We show here that expression of RhoA, ROCK1 and ROCK2 were higher in malignant T4-2 cells compared to the counterpart nonmalignant S1 cells (Figure 1). These results are similar to observations in clinical tissue samples [9–13], indicating that culture of HMT-3522 breast cancer progression series in 3D lrECM gels is a good model for studying the more detailed mechanism(s) of how these pathways may disrupt normal function. We show that phosphorylation of MLC, a substrate of ROCK, was increased in T4-2 cells (Figure 1). We previously showed that the cytoskeleton is disorganized, and that there is high mRNA expression of actin cytoskeleton genes in T4-2 cells in 3D lrECM [22–25]. These results suggested activation of RhoA/ROCK pathway in T4-2 cells. Inhibition of ROCK by two different ROCK inhibitors improved the basal and basolateral polarity (Figure 3B and 3C), and inhibited proliferation (Figure 2B) of T4-2 cells. Moreover, inhibition of ROCK led to down-regulation of EGFR and Integrinβ1 signaling pathways [22, 23], and reduced expression of GLUT3 and LDHA (Figure 2C and Figure 5A), which are involved in aerobic glycolysis [24]. Moreover, inhibition of ROCK suppressed the proliferation in T4-2 cells co-cultured with lung fibroblasts in IrECM (Figure 7). ROCK inhibition partially restored basal polarity, inhibited cell proliferation and down-regulated Integrinβ1 and GLUT3 in metastatic MDA-MB-231 cells grown in 3D cultures of lrECM (Figure 9). These observations are consistent with those reported where inhibition of ROCK suppresses tumor formation in an orthotopic MDA-MB-231 xenograft mouse model [16]. In contrast, nonmalignant cells were only slightly affected by treatment with ROCK inhibitors (Figure S2) [43]. However, forced expression of active ROCK disrupted polarity in nonmalignant cells (Figure 6). Hence, our results indicate that improper regulation of RhoA/ROCK signaling leading to cytoskeletal rearrangement plays an important role in regulation of growth and polarity in cancer cells in 3D.

Culture of cells under physiologically relevant 3D conditions distinguishes the morphology between nonmalignant (normal) cells and cancer cells, [19–21]. Using this culture system, we found expression of ROCK1 and ROCK2 were high in malignant T4-2 cells in 3D lrECM, but *not* in 2D cultures (Figure 1). We also observed reciprocal interactions among ROCK, EGFR, Integrinβ1 and aerobic glycolysis pathways (Figure 2C and Figure 5). Importantly, the interaction was dependent on the 3D context. Although ROCK inhibitors reduced actin-stress fibers in T4-2 cells cultured in 2D, they failed to down-regulate expression of EGFR, Integrinβ1 and GLUT3 proteins (Figure S1). This is consistent with previous results from the laboratory where we found that cross-modulation between EGFR and Integrinβ1 signaling pathways occurred only in 3D culture, but not in 2D culture [23]. Moreover, a mouse skin tumor model where cells expressed 4-hydroxytamoxifen-regulated ROCK, which consists of ROCK kinase domain and estrogen receptor under control of cytokeratin 14 promoter [44], revealed a relationship between ROCK-induced cytoskeletal rearrangement and tumor progression [44]. Phosphorylated AKT, phosphorylated FAK and β-catenin mislocalization were elevated in the ROCK-activated skin tissues while ROCK inhibitor blocked the phosorylation. Additionally, these studies showed that activation of ROCK increased the tissue stiffness, accompanied by ECM decomposition [44]. Similarity, our 3D culture model showed that phosphorylated AKT, phosphorylated FAK (Figure 5B) and disorganized β-catenin localization (Figure 3C) were increased in malignant T4-2 cells compared to nonmalignant S1 cells. Inhibition of ROCK reduced levels of phosphorylated AKT, phosphorylated FAK and disorganized β-catenin in T4-2 cells in 3D lrECM (Figure 3C and 5B). Our studies further support the use of physiological 3D culture as a platform to understand the integrated signaling required to form correct tissue polarity and what goes wrong during cancer progression. Another group has developed 3D cultures for glioblastoma using polyethylene-glycol-based hydrogel to modulate matrix stiffness, and found that increasing matrix stiffness induced upregulation of ROCK1 [45]. Therefore, it is worth investigating using other type of cancer cells in 3D culture.

The detail mechanism of upregulation of ROCK expression has not been fully elucidated in breast cancer. ROCK1 and ROCK2 are ubiquitously expressed during embryogenesis and in adult tissues [5, 6]. Comparatively, ROCK1 is highly expressed in the lung, liver, spleen, kidney and testis while ROCK2 is abundant in the brain and heart [5, 6]. However, regulation of expression of ROCK1 and ROCK2 is often abrogated in several diseases [5, 6, 46], including several cancers [9, 11, 12, 36–40]. More recently, hypoxia-inducible factor (HIF) has been shown to transcriptionally up-regulate RhoA and ROCK1, not but ROCK2, in breast cancer cell lines in response to hypoxia in 2D culture. In previous work from the laboratory, it was observed that siRNA-mediated knockdown of HIF-1α, HIF-2α or their combination neither down-regulated EGFR and Integrinβ1 nor revert the disorganized phenotype of T4-2 cells [24]. In the current study, both ROCK1 and ROCK2 were increased in 3D lrECM of T4-2 cells (Figure 1), despite the fact that HIF is not involved. Thus, there is little possibility of involvement of HIF in regulation of ROCK expression in the 3D culture system and most probably even *in vivo*.

There are several reports to illustrate the decrease in tumor suppressive microRNAs which directly regulate expression of ROCK1 and ROCK2 in other type of cancers [47–53]. We are now finding that several microRNAs were decreased in T4-2 cells and MDA-MB-231 compared with S1 cells, and their microRNAs were also decreased in breast tumor tissues (submitted manuscript). It is likely that microRNAs directly or indirectly regulate ROCK mRNA expression in breast cancer in the similar fashion as other types of cancers.

To confirm the commonality of ROCK inhibition in attenuating the breast cancer phenotype, we show here that inhibition of ROCK restored polarity, and down-regulated the expression of Integrinβ1 and GLUT3 proteins in MDA-MB-231 cells (Figure 9) as well as T4-2 cells. On the other hand, ROCK inhibition had a much less effect on MDA-MB-231 cell proliferation of MDA-MB-231 as compared to T4-2 cells (Figure 9B). MDA-MB-231 cells have lower levels of EGFR in comparison to some breast cancer cell lines (Figure 8B) which leads to the ineffectiveness of EGFR inhibitors (Figure 8B) and EGFR function blocking antibody as shown previously [26]. This is because cell growth of MDA-MB-231 is driven by mutated K-RAS (G13D) rather than by EGFR [54–56]. MDA-MB-231 cells have a large amount of ROCK1 and ROCK2, regardless of 2D or 3D culture condition (Figure 8B). Furthermore, a major difference between MDA-MB-231 and T4-2 cells is that MDA-MB-231 lacks E-cadherin (Figure 8B). Thus, these differences most likely account for the modest inhibition in proliferation by the ROCK inhibitor on MDA-MB-231 cells.

Another faulty signaling pathway in MDA-MB-231 cells is the lack of functional E-cadherin and the loss of E-cadherin in cells is associated with epithelial-mesenchymal transition (EMT), accompanied by the potential of drug resistance in cancer treatment [57, 58]. ROCK inhibition decreased proliferation, and enhanced apoptosis in MDA-MB-231 cells overexpressing E-cadherin (Figure 10D). Thus, one explanation is that inhibition of EMT by overexpression of E-cadherin might contribute to enhancement of responsiveness to ROCK inhibitor. It has recently been shown that ROCK1 is associated with E-cadherin complexes through p120-catenin [59]. Our above observations also indicate that there is a close relationship between ROCK and E-cadherin.

Inhibition of ROCK leading to apoptosis is unclear and there have been reports that ROCK positively or negatively regulates apoptosis. Capase-2/3 and granzyme B cleave ROCK1 and ROCK2, respectively, at C-terminal region of the protein during apoptosis [60–62]. In consequence, each ROCK is activated, leading to apoptotic membrane blebbing [60–62]. The other apoptotic biochemical reactions such as condensation of chromatin and inactivation of mitochondrial enzymes are independent of membrane blebbing *via* ROCK [63]. On the other hand, inhibition of ROCK induces apoptosis in several types of cancer cell lines [64, 65], and enhances cisplatin-induced apoptosis in lung cancer line [66]. Combined therapy of both inhibition of ROCK by Fasudil and the proteasome complex by bortezomib induces apoptosis, and drastically reduces lung tumor burden in a genetic engineered mouse model harboring KRAS mutation without adverse side effects [67]. We observed here that induction of apoptosis by inhibition of ROCK took place in MDA-MB-231 cells overexpressing E-cadherin, not but in T4-2 cells, S1 cells or MDA-MB-231 vector control cells. Therefore, the role of ROCK in a pro-apoptotic process might depend on a cell type or intracellular context. The precise mechanism of inhibition of ROCK leading to apoptosis requires further investigation.

In conclusion, our findings indicate that RhoA/ROCK signaling, which leads to cytoskeletal rearrangement, plays an important role in the phenotype of breast cancer cells and the disrupted tissue architecture in breast cancer. Therapy based on targeting the ROCK signaling cascade or combinational therapy with inhibitors/blocking antibodies against other pathways might provide a therapeutic opportunity for breast cancer.

## MATERIALS AND METHODS

### Cell culture

HMT-3522 mammary epithelial cells were cultured in H14 medium (DMEM/F12 containing insulin at 250 ng/mL, transferrin at 10 μg/mL, sodium selenite at 2.6 ng/mL, 0.1 nM estradiol, 1.4 μM hydrocortisone, and prolactin at 5 μg/mL) [22–25]. The nonmalignant S1 cells were grown on dishes in the H14 medium containing EGF at 10 ng/mL, and the malignant T4-2 cells were propagated on collagen type I-coated dishes in the absence of EGF. MCF10A cells were cultured in DMEM-F12 containing horse serum at 5%, hydrocortisone at 0.5 mg/ml, insulin at 10 μg/ml, EGF at 20 ng/ml, Cholera Toxin at 100 ng/mL and penicillin/streptomycin. As for overexpression of constitutively active ROCK, MCF10A cells were transiently transfected with pCAG-myc-tagged (pCAG-myc) ROCKΔ3 using Fugene HD (Roche). Under this condition, we confirmed that transfection efficiency was 20~30% using a plasmid expressing EGFP. MDA-MB-231 cells were obtained from the American Type Culture Collection. MDA-MB-231 cells were cultured in DMEM/F12 supplemented with 10% fetal bovine serum and penicillin/streptomycin. E-cadherin-expressing MDA-MB-231 cells were previously established by transfection of parental MDA-MB-231 cells with a full-length mouse E-cadherin cDNA in pBATEM2 plasmid under the control of the chicken-actin promoter [26]. E-cadherin and vector-expressing MDA-MB-231 cells were DMEM/F12 supplemented with 10% fetal bovine serum, penicillin/streptomycin and 500 μg/mL G418. Cultures were maintained under 5% CO_2_ at 37°C. 3D lrECM on-top cultures were prepared by trypsinization of cells from tissue culture plastic, seeding of single cells on top of a thin gel of Engelbreth-Holm-Swarm (EHS) tumor extract (Growth Factor Reduced Matrigel: BD Biosciences), and addition of medium containing 5% EHS [19]. Cells were maintained by changing their propagation medium every 2 days. S1, MCF10A, T4-2 and MDA-MB-231 cells were cultured for 5-6 days, 6-7 days, 7-8 days and 7-8 days, respectively.

### Chemical inhibitors and blocking antibodies

The ROCK inhibitors Fasudil hydrochloride, Y-27632 dihydrochloride and GSK-429286 were obtained from Tocris. The following materials were purchased from the indicated commercial sources: PD98059 (MEK inhibitor, New England Biolabs); AG1478 (EGFR inhibitor, Calbiochem); AIIB2 (anti-Integrinβ1 antibody; Aragen Bioscience); mAb225 (anti-EGFR antibody; Oncogene); Blebbistatin (myosin II ATPase inhibitor, Cayman Chemical).

### Western blot analysis

Cells were isolated from 3D culture with PBS/EDTA as previously described [22–25]. The cells were lysed with ice-cold RIPA buffer (20 mM Tris-HCl pH 7.5, 150 mM NaCl, 1 mM Na_2_EDTA, 1 mM EGTA 1% NP-40, 1% sodium deoxycholate, 2.5 mM sodium pyrophosphate, 1 mM β-glycerophosphate, 1 mM Na_3_VO_4_, 1 μg/ml leupeptin, 1 mM PMSF) supplemented with protease inhibitor cocktail set I (Calbiochem) and phosphatase inhibitor cocktail set (Calbiochem), and then sonicated on ice. Insoluble materials were removed by centrifugation and the supernatant was boiled with Laemmli's sample buffer containing dithiothreitol for 5 min. Equivalent amounts of protein in each sample (10 μg/lane) were separated by SDS-polyacrylamide gel electrophoresis, and then blotted onto a PVDF membrane (Immobilon, Millipore). Proteins were detected using an immunoblotting technique with the following specific antibodies: anti-ROCK1 antibody (Cell Signaling Technology); anti-ROCK2 antibody (Cell Signaling Technology); anti-RhoA antibody (Cell Signaling Technology); anti-MLC antibody (Cell Signaling Technology); anti-phospho-MLC (Ser19) antibody (Cell Signaling Technology); anti-Integrinβ1 antibody (Chemicon); anti-EGFR antibody (Chemicon); anti-Lamin A/C antibody (Santa Cruz Biotechnology); anti-E-cadherin antibody (BD phamingen); anti-GLUT3 antibody (GeneTex); LDHA (Santa Cruz Biotechnology); anti-Akt antibody (Cell Signaling Technology); anti-phospho-Akt (Ser473) antibody (Cell Signaling Technology); anti-p44/42 MAP kinase (ERK1/2) antibody (Cell Signaling Technology); anti-phospho-p44/42 MAP kinase (Thr202/Tyr204) antibody (Cell Signaling Technology); anti-FAK antibody (Cell Signaling Technology); anti-phospho-FAK (Tyr925) antibody (Cell Signaling Technology); Horseradish peroxidase-linked whole antibody (Amersham Bioscience) was used as a secondary antibody. The antibodies were detected with SuperSignal West Femto chemiluminescence reagent (Pierce) and analyzed using FluorChem HD2 Imaging System (Cell Biosciences) or FluorChem 8900 imager (Alpha Innotech).

### Real-Time quantitative reverse transcription PCR (RT-PCR)

Total RNA was isolated on-column DNaseI digestion according to the manufacturer's instruction (RNeasy). First strand-cDNA synthesis was performed from 0.5-1.5 μg of total RNA in 20-μL volumes with oligo (dT) priming using the Superscript First-strand Synthesis System (Invitrogen). Quantitative real-time PCR analysis was performed with the Lightcycler System using the LightCycler 480 SYBR I Green Maser (Roche). The Lightcycler PCR amplification protocol was as followed: 95°C for 5 min (initial denaturation), and 45 amplification cycles (95°C for 10 s, 60°C for 60s), 50°C for 10 sec (cooling). Amplification was followed by melting curve analysis to verify the presence of a single PCR product. The following primer pairs were synthesized by Integrated DNA Technologies: ROCK1, 5′-gatcacactgttagtcggcttg-3′ and 5′-atctcctcctccttctccagtt-3′; ROCK2, 5′-gattctgtatgccaatgaaggag-3′ and 5′-ctcacagttggttgggaaatg-3′; TATA binding protein (TBP), 5′-taatcccaagcggtttgct-3′ and 5′-ctgttcttcactcttggctcct-3′. mRNA expression levels of ROCK1 and ROCK2 were normalized to that of TBP.

### Microarray data

Microarray analysis has been previously performed using the Affymetrix high-density oligonucleotide array human HG-U133A microarrays (platforms GPL3921 and GPL4685) [25]. Microarray data have been deposited in GEO (Accession no. GSE50444).

### Immunofluorescence

Colonies in 3D lrECM gel were smeared on glass slides. After brief air-drying, colonies were fixed with 3.7% paraformaldehyde in PBS at room temperature for 20 min and were washed with PBS containing 0.1 mM glycine. After blocking with immunofluorescence buffer (0.2% Triton X-100, 0.05% Tween 20, 0.1% BSA, 7.7 mM NaN_3_ in PBS) containing 10% goat serum and 1% goat F(ab')2 anti-mouse immunoglobulin G (IgG; Caltag) at room temperature for 30 min, colonies were stained with anti-Integrinα6 antibody (BD phamingen) or anti-β-catenin antibody (BD phamingen) at 4°C overnight. Colonies were washed with immunofluorescence buffer, and stained with Alexa Fluoro 488-labeled anti-IgG antibody (Invitogen) at room temperature for 2 hr. Colonies were washed with immunofluorescence buffer and PBS, and nuclei were stained with 4′,6-Diamidino-2-phenylindole (DAPI, Sigma-Aldrich) at room temperature for 5 min. Mounting medium was dropped and gently lay down coverslip, and the glass slides were stored at −20°C before imaging analysis. Fluorescent images were acquired using Zeiss LSM710 Meta confocal microscope.

### F-actin stain

Colonies in 3D lrECM gel were smeared on glass slides. After fixed with 3.7% paraformaldehyde for 20 min, colonies were permeabilized with 0.1% Triton X-100 in PBS for 5 min, and blocked with 0.1% BSA at room temperature for 30 min. Colonies were stained with Alexa Fluor 633 phalloidin (invtirogen) for 30 min, and washed with PBS and subsequently stained with DAPI. Fluorescent images were acquired using Zeiss LSM710 Meta confocal microscope.

### TdT-mediated dUTP nick end labeling (TUNEL) stain

Colonies in 3D lrECM gel were smeared on glass slides. After fixed with 3.7% paraformaldehyde for 60 min, colonies were permeabilized with 0.1% Triton X-100 in 0.1% sodium citrate for 5 min, and washed with PBS. Colonies were stained with TUNEL reaction mixture (Roche, *In situ* cell death detection kit) at 37°C 70 min and were washed with PBS. Nuclei were subsequently stained with DAPI. Fluorescent images were acquired using Zeiss LSM710 Meta confocal microscope.

### Proliferation assay

Mitochondrial dehydrogenase activity was determined by cleavage of 3-(4,5-dimethylthiazol-2-yl)-2,5-diphenyl tetrazolium bromide (MTT, Sigma-Aldrich) to purple formazan as an index of cell viability. 5 mg/mL MTT was added to the 3D lrECM gel culture. After a 4h-incubation, the generated formazan crystals were dissolved with 10% SDS containing 0.04 N HCl at 37°C overnight. The solubilized formazan product was measured at 590 nm using an automatic microtiter plate reader (SpectraMax, Molecular Probes).

### Co-culture of T4-2 cells with fibroblasts

Yellow fluorescent Protein (YFP)-T4-2 cells were established by infection with pLentiCMV/YFP lentivirus. Primary human lung fibroblasts were obtained from Lonza and cultured in high-glucose DMEM supplemented with 10% fetal bovine serum and penicillin/streptomycin. Primary lung fibroblasts were seeded in 96 well-plate at a concentration of 5 × 10^4^ cells in 100 μl of culture medium. Plate was left undisturbed on a flat surface for 20 min to allow even cell seeding before incubation. After 2 days, YFP-T4-2 cells were suspended in unsupplemented DMEM/F12. After carefully washing cultures three times with PBS, YFP-T4-2 cells were seeded at a concentration of 10^2^ cells per well in 100 μl. Cells were allowed to settle for 15 min at room temperature, and then a drip of 10% EHS in 100 μl unsupplemented DMEM/F12 was slowly added to each well. After 12~15 days of co-culture, the objective was centered to each well before acquisition of 6 × 6 tiles which captured the entirety of each well and then the brightness of YFP per well was measured by Zeiss LSM710 Meta confocal microscope.

### Statistical analysis

The Student's *t*-test following the F-test was used for analysis of differences between two groups. Multiple comparisons among treatment groups were assessed by one-way analysis of variance, followed by the Dunnett's test. Values of *P* < 0.05 were considered statistically significant.

## SUPPLEMENTARY MATERIAL FIGURES


