# Supplementary material for: Inhibitors of Rho kinase (ROCK) signaling revert the malignant phenotype of breast cancer cells in 3D context
Source: Oncotarget. 2016 May 17;7(22):31602–22. doi: 10.18632/oncotarget.9395 (PMC5077963; doi:10.18632/oncotarget.9395)
Supplement: Supplementary file 1 [file oncotarget-07-31602-s001.pdf]

Inhibitors of Rho kinase (ROCK) signaling revert the malignant phenotype of breast cancer cells in 3D context

Supplementary Material

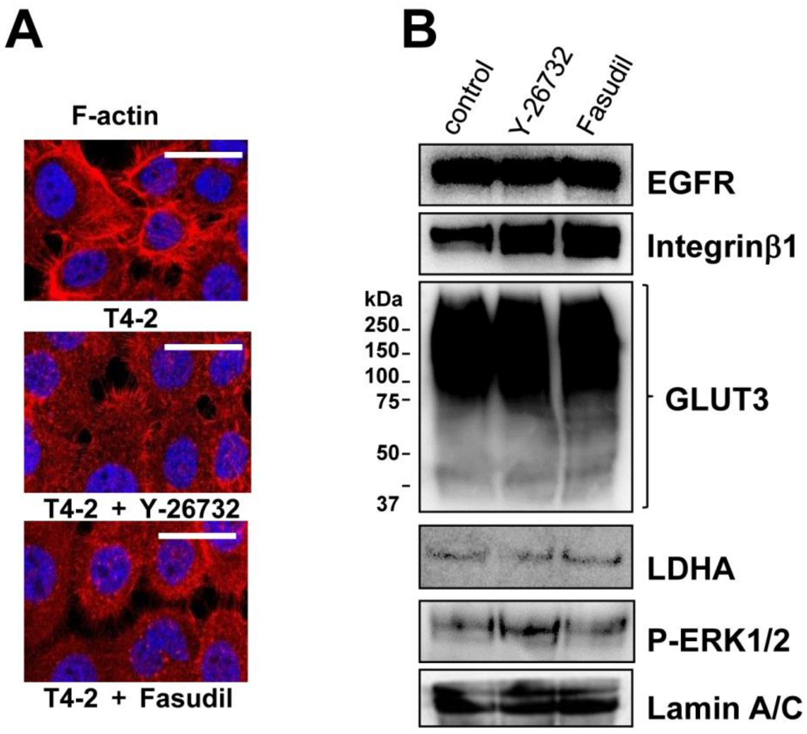

Fig. S1. Absence of down-regulation by inhibition of ROCK in T4-2 cells in monolayer culture. (A) Confocal immunofluorescence of F-actin in T4-2 cells treated with ROCK inhibitor (Y-26732 or Fasudil, 30 μM). Images were obtained using Alexa Fluor-conjugated phalloidin (F-actin; Red, Nuclei; Blue). Scale bars: 10 μm. (B) Protein expression of EGFR, Integrinβ1, GLUT3, LDHA, P-ERK1/2 and Lamin A/C. Total cell lysates were analyzed by Western blotting with their specific antibodies.

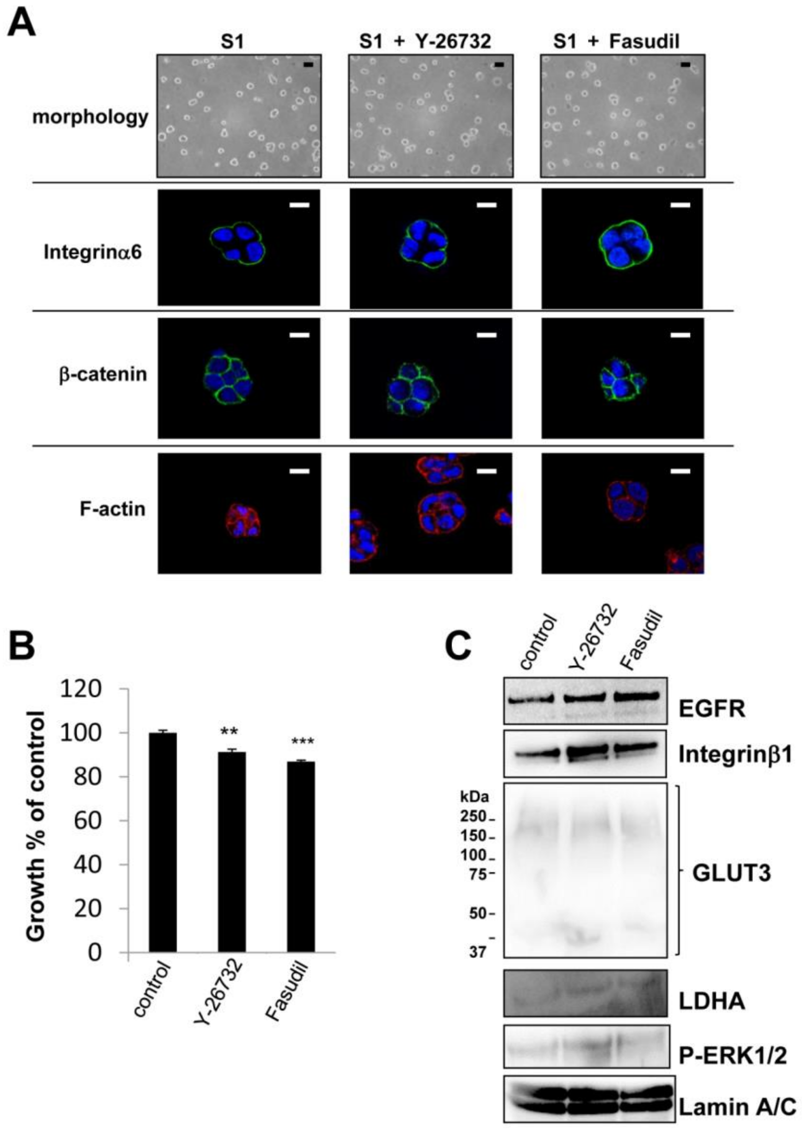

Fig. S2. Effects of inhibition of ROCK on nonmalignant S1 cells in 3D lrECM. (A) Morphological alternations and confocal immunofluorescence images of S1 cells treated with ROCK inhibitor (Y-26732 or Fasudil, 30 μM). Their images were obtained using their specific antibodies or Alexa Fluor-conjugated phalloidin (Integrinα6; Green, β-catenin; Green, F-actin; Red, Nuclei; Blue). Black scale bars: 20 μm, White scale bars: 10 μm. (B) Proliferation of S1 cells treated with ROCK inhibitor (Y-26732 or Fasudil, 30 μM). Cell viability was assessed by MTT assay. Values represent means ± SE of four determinations. **P<0.01, ***P<0.001 compared with vehicle control group (Student's t). (C) Protein expression of EGFR, Integrinβ1, GLUT3, LDHA, P-ERK1/2, Lamin A/C. Total cell lysates were analyzed by Western blotting with their specific antibodies.

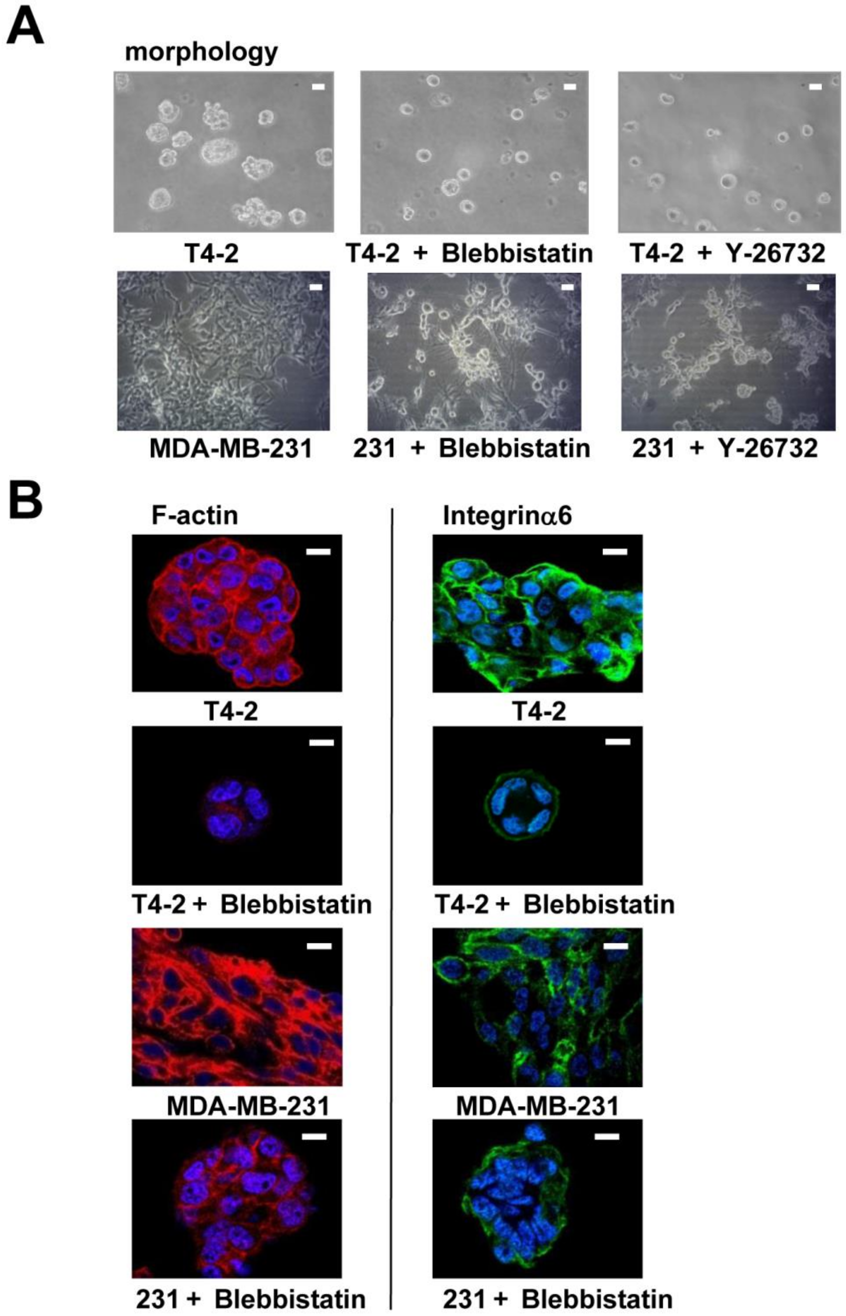

Fig. S3. Effects of Blebbistatin, a Myosin II ATPase inhibitor, on T4-2 or MDA-MB-231 cells in 3D lrECM. (A) Morphological alternations and (B) confocal immunofluorescence images of T4-2 or MDA-MB-231 cells treated with Blebbistatin (20 μM) or Y-26732 (30 μM). Their images were obtained using anti-integrinα6 antibody or Alexa Fluor-conjugated phalloidin (integrinα6; Green, F-actin; Red, Nuclei; Blue). Scale bars: 20 μm (A), 10 μm. (B).

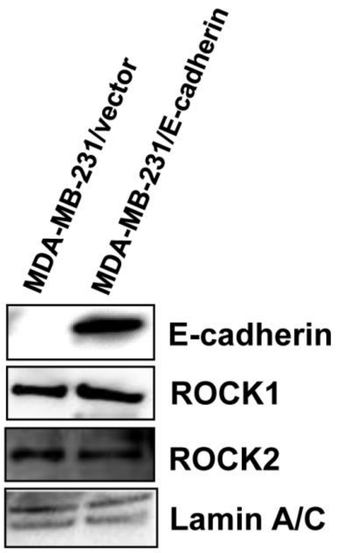

Fig. S4. Protein expression of E-cadherin, ROCK1, ROCK2 and Lamin A/C in vector-or E-cadherin-expressing MDA-MB-231 cells. Total cell lysates were analyzed by Western blotting with their specific antibodies.
